# Supplementary material for: Causal Relationship between Aging and Anorexia Nervosa: A White-Matter-Microstructure-Mediated Mendelian Randomization Analysis
Source: Biomedicines. 2024 Aug 16;12(8):1874. doi: 10.3390/biomedicines12081874 (PMC11351342; doi:10.3390/biomedicines12081874)
Supplement: Supplementary file 1 [file biomedicines-12-01874-s001.zip › Supplementary Figure/Figure S4/Singe SNP plot of MR analysis for TL to ubm-b-1580.pdf]

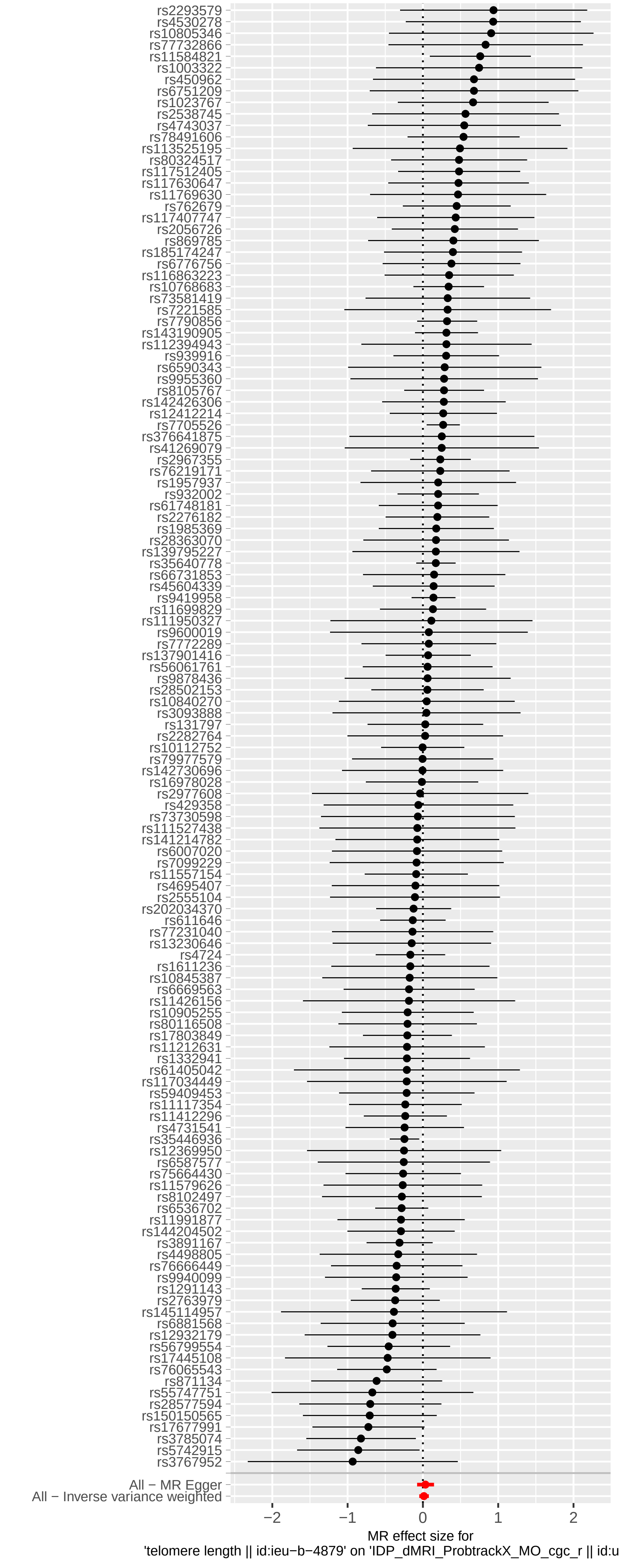
rs2293579
rs4530278
rs10805346
rs77732866
rs11584821
rs1003322
rs450962
rs6751209
rs1023767
rs2538745
rs4743037
rs78491606
rs113525195
rs80324517
rs117512405
rs117630647
rs11769630
rs762679
rs117407747
rs2056726
rs869785
rs185174247
rs6776756
rs116863223
rs10768683
rs73581419
rs7221585
rs7790856
rs143190905
rs112394943
rs939916
rs6590343
rs9955360
rs8105767
rs142426306
rs12412214
rs7705526
rs376641875
rs41269079
rs2967355
rs76219171
rs1957937
rs932002
rs61748181
rs2276182
rs1985369
rs28363070
rs139795227
rs35640778
rs66731853
rs45604339
rs9419958
rs11699829
rs111950327
rs9600019
rs7772289
rs137901416
rs56061761
rs9878436
rs28502153
rs10840270
rs3093888
rs131797
rs2282764
rs10112752
rs79977579
rs142730696
rs16978028
rs2977608
rs429358
rs73730598
rs111527438
rs141214782
rs6007020
rs7099229
rs11557154
rs4695407
rs2555104
rs202034370
rs611646
rs77231040
rs13230646
rs4724
rs1611236
rs10845387
rs6669563
rs11426156
rs10905255
rs80116508
rs17803849
rs11212631
rs1332941
rs61405042
rs117034449
rs59409453
rs11117354
rs11412296
rs4731541
rs35446936
rs12369950
rs6587577
rs75664430
rs11579626
rs8102497
rs6536702
rs11991877
rs144204502
rs3891167
rs4498805
rs76666449
rs9940099
rs1291143
rs2763979
rs145114957
rs6881568
rs12932179
rs56799554
rs17445108
rs76065543
rs871134
rs55747751
rs28577594
rs150150565
rs17677991
rs3785074
rs5742915
rs3767952
All − MR Egger
All − Inverse variance weighted
−2
−1
0
1
2
MR effect size for
'telomere length || id:ieu−b−4879' on 'IDP_dMRI_ProbtrackX_MO_cgc_r || id:u
